# A germline TaqI restriction fragment length polymorphism in the progesterone receptor gene in ovarian carcinoma.

**DOI:** 10.1038/bjc.1995.92

**Published:** 1995-03

**Authors:** N. J. McKenna, D. G. Kieback, D. N. Carney, M. Fanning, J. McLinden, D. R. Headon

**Affiliations:** Department of Biochemistry, University College Galway, Ireland.

## Abstract

**Images:**


British Journal of Cancer (1995) 71, 451-455

© 1995 Stockton Press All rights reserved 0007-0920/95 $9.00

### A germline *Taq*I restriction fragment length polymorphism in the progesterone receptor gene in ovarian carcinoma

NJ McKenna[1], DG Kieback[2]*, DN Carney[3], M Fanning[3], J McLinden[4] and DR Headon[1]

[1]*Cell and Molecular Biology Group, Department of Biochemistry, University College Galway, Galway, Ireland;* [2]*Department of Obstetrics and Gynaecology, University of Ulm, Prittwitzstraße 43, D-89075 Ulm, Germany;* [3]*Department of Oncology, Mater Misericordiae Hospital, Eccles St., Dublin 7, Ireland;* [4]*American Biogenetic Sciences, South Bend, Notre Dame, Indiana 46556, USA.*

**Summary** Clinical outcome in ovarian carcinoma is predicted by progesterone receptor status, indicating an endocrine aspect to this disease. Peripheral leucocyte genomic DNAs were obtained from 41 patients with primary ovarian carcinoma and 83 controls from Ireland, as well as from 26 primary ovarian carcinoma patients and 101 controls in Germany. Southern analysis using a human progesterone receptor (hPR) cDNA probe identified a germline *Taq*I restriction fragment length polymorphism (RFLP) defined by two alleles: T1, represented by a 2.7 kb fragment; and T2, represented by a 1.9 kb fragment and characterised by an additional *Taq*I restriction site with respect to T1. An over-representation of T2 in ovarian cancer patients compared with controls in the pooled Irish/German population ($P<0.025$) was observed. A difference ($P<0.02$) in the distribution of the RFLP genotypes between Irish and German control populations was also observed. The allele distributions could not be shown to differ significantly from Hardy-Weinberg distribution in any subgroup. Using hPR cDNA region-specific probes, the extra *Taq*I restriction site was mapped to intron G of the hPR gene.

**Keywords**: progesterone receptor gene; RFLP; ovarian carcinoma

Ovarian cancer is the leading cause of death among patients with gynaecological cancers with a 5 year survival rate, averaged over all stages, of 29-32% (Scully, 1987). Such high mortality is due largely to the insidious early progression of the disease and consequent detection at an advanced tumour stage. The absence of a sufficiently specific and sensitive screening test for diagnosis or prognosis of the disease compounds the problem of its treatment.

Progesterone receptor estimation yields prognostic information in ovarian carcinoma (Slotman *et al.*, 1990; Chadha *et al.*, 1993; Noguchi *et al.*, 1993), indicating a positive relationship between progesterone receptor expression and prognosis. The cDNA for the human progesterone receptor (hPR) was first cloned and sequenced by Misrahi *et al.* (1987) and later by Kastner *et al.* (1990). The structure of the hPR gene has recently been described (Figure 1a) (Misrahi *et al.*, 1993). The presence of multiple forms of the hPR in ovarian carcinoma has been reported (Scharl *et al.*, 1991). Loss of heterozygosity at the 11q23 locus in ovarian carcinoma has been reported (Foulkes *et al.*, 1993), indicating the presence in this region of a tumour-suppressor gene relevant to ovarian carcinoma. The hPR gene has been mapped by separate groups to the 11q22-q23 locus (Rousseau-Merck *et al.*, 1987; Mattei *et al.*, 1988). Transfection of an ovarian carcinoma cell line with chromosome 11 has resulted in suppression of growth (Cao *et al.*, 1993), further suggestive of the presence of tumour-suppressive regions on this chromosome.

Restriction fragment length polymorphisms (RFLPs) are somatic or hereditary variations in the length of a DNA fragment yielded by specific restriction endonuclease digestion. Steroid hormone receptor gene RFLPs have been previously studied in human female malignancies. A *Hind*III polymorphism in the progesterone receptor gene exhibited non-Mendelian distribution in primary breast tumours (Fuqua *et al.*, 1991). A *Hind*III polymorphism of the oestrogen receptor gene was found to correlate with progesterone receptor expression in primary breast tumours (Wanless *et al.*, 1991). Furthermore, a specific pattern of differential expression of the chicken ovalbumin upstream promoter transcription factor (COUP-TF), an orphan of the steroid hormone receptor superfamily, has been identified in human ovarian cancer cell lines (Kieback *et al.*, 1993), indicating the possible role of this novel steroid receptor in ovarian carcinoma.

The *Taq*I restriction endonuclease recognition site TCGA, containing all four base pairs found in DNA, allows analysis of all 12 potential single base pair mutations (Sandy *et al.*, 1992). The presence in this recognition site of the dinucleotide sequence CpG is of particular importance given that deamination of cytosine or 5-methylcytosine in CpG dinucleotides is a common mechanism for the formation of C to T transitions in cancer-related genes (Hollstein *et al.*, 1991). For these reasons, *Taq*I is especially useful in discerning polymorphic alleles by RFLP analysis.

In order to investigate the status of the hPR gene in primary ovarian carcinoma, we analysed peripheral leucocyte genomic DNA from ovarian carcinoma patients and controls for appearance of a germline *Taq*I RFLP in the hPR gene.

### Materials and methods

### *Polymerase chain reaction (PCR) and hPR cDNA region-specific probes*

Refer to Figure 1. Screening of a λgt11 random-primed T47D human breast cancer cell line cDNA library with a 2 kb chick oviduct progesterone receptor cDNA (both gifts from Dr BW O'Malley, Baylor College of Medicine, Houston, TX, USA) yielded a 1.85 kb cDNA, hPR-1, which was subsequently cloned in pGEM-4 (Promega, Madison, WI, USA) using *Eco*RI to yield pGEM-4-hPR-1 (Clifford-Brougham, 1989). The hPR-1 cDNA (Figure 1b) consists of 1846 base pairs (bp), of which 1084 bp represent 3' coding sequence and the remaining 762 bp is 3'-untranslated sequence. hPR-1 probe was prepared for Southern analysis by digestion of pGEM-4-hPR-1 with *Eco*RI (Boehringer Mannheim), under conditions specified by the manufacturer, and purified twice by electroelution after separation on a 1% agarose electrophoresis gel. The hPR-2 cDNA probe (Figure 1c) was generated by PCR using sense primer no. 1 (5'-TCGAGCTC-

Correspondence: DR Headon
*Present address: Department of Obstetrics and Gynaecology, Baylor College of Medicine, Houston, Texas 77030, USA.
Received 22 September 1994; revised 31 October 1994; accepted 1 November 1994

**Figure 1** (a) Structure of the human progesterone receptor (hPR) gene (Misrahi *et al*., 1993). Numbers above the exons (E1–E8) refer to cDNA base pair number (numbered from ATG1). T, location of *Taq*I cDNA restriction sites according to Kastner *et al*. (1990). Introns (A–G) are represented by broken thin lines. The locations of the translation initiation (ATG) and translation termination (TGA) codons are indicated. The hPR DNA-binding domain is encoded by exons 2 and 3. Exon 4 encodes the hinge domain and part of the hormone-binding domain. Exons 5–8 encode the remainder of the hPR hormone-binding domain (Misrahi *et al*., 1993). The cDNA probes used in Southern analysis (b) hPR-1, (c) hPR-2 and (d) hPR-3, are shown.

ACAGCGTTTCTATCAA-3′) corresponding to hPR cDNA sequence 2593–2616 bp (numbered from ATG1), and the antisense primer no. 2 (5′-AAAACCTACAAAACCCACAA-TACT-3′) (hPR cDNA sequence 3471–3494 bp, numbered from ATG1) (Kastner *et al*., 1990). hPR-3 probe (Figure 1d) was generated by PCR amplification using the sense primer no. 3 (5′-TCGACTTAAGAGAAAAAATCTTAC-3′) (hPR cDNA sequence 3133–3156 bp, numbered from ATG1) (Kastner *et al*., 1990), and primer no. 2. Polymerase chain reactions (PCRs) were carried out in a 100 μl final volume of 10 mM Tris–HCl, 1.5 mM magnesium chloride and 50 mM potassium chloride using 50 ng each of the oligonucleotide primer pair, 100 ng of template pGem-4-hPR-1, 1.5 mM of each dNTP and 2.5 units of *Taq* DNA polymerase (all PCR reagents from Boehringer Mannheim). Each amplification cycle (30 in total) consisted of a 90 s denaturation step at 94°C, a 90 s annealing step at 55°C and a 90 s extension step at 72°C. PCR products were purified on a 1% low-melt agarose gel (NuSieve GTG; FMC Bioproducts, Rockland, ME, USA) before nick translation $^{32}$P-labelling.

*DNA extraction, restriction enzyme digestion and Southern blot analysis*

Genomic DNA was isolated from whole human blood by a modification of the method of Miller *et al*. (1988). Thawed whole blood was incubated with an equal volume of 10 mM Tris–HCl–10 mM EDTA (pH 8.0) and centrifuged at 5000 r.p.m. for 10 min at 4°C. The supernatant was removed and the pellet resuspended in 10 mM Tris–HCl–10 mM EDTA (pH 8.0) and centrifuged at 5000 r.p.m. for 10 min. The supernatant was removed and the pellet washed in 10 mM Tris–HCl– 10 mM EDTA (pH 8.0). After centrifugation at 5000 r.p.m. for 10 min at 4°C, the pellet was resuspended in 10 mM Tris–HCl pH 8.0–5 mM EDTA–1% sodium dodecyl sulphate (SDS)–0.4% Proteinase K (Sigma, St Louis, MO, USA), incubated at 37°C overnight, and treated as described previously (Miller *et al*., 1988). After spectrophotometric determination of DNA concentration and purity, 20 μg of each DNA was digested to completion with 40 units of *Taq*I (Boehringer Mannheim) in a volume of 300 μl at 65°C overnight under conditions specified by the manufacturer. The digested DNA samples were fractionated on a 0.8% agarose gel and following denaturation (1.5 M sodium chloride, 0.5 M sodium hydroxide) and neutralisation (1.5 M sodium chloride, 0.5 M Tris pH 7.2, 0.001 M EDTA) DNA was transferred from the gel in 20 × SSC (3 M sodium chloride, 0.3 M sodium citrate) by capillary action to a 6 × SSC-soaked nylon membrane (Boehringer Mannheim). After overnight transfer of DNA, the membranes were washed in 2 × SSC and baked for 2 h at 80°C. Low-melt agarose gel-purified probe was $^{32}$P-labelled using Prime-A-Gene (Promega, Madison, WI, USA) to a specific activity of $2 \times 10^8$ c.p.m. $\mu g^{-1}$. Prehybridisation was carried out in 6 × SSC, 5 × Denhardt's solution [0.02% bovine serum albumin, 0.02% polyvinylpyrollidone, 0.02% Ficoll (Pharmacia, Uppsala, Sweden)], 0.5% SDS and 100 $\mu g\ ml^{-1}$ denatured salmon sperm DNA at 65°C for 3 h. The membranes were incubated in hybridisation fluid (as prehybridisation fluid, containing $5 \times 10^6$ c.p.m. $^{32}$P labelled probe) at 65°C for 18–24 h. Membranes were washed to a final stringency of 0.5 × SSC–0.5% SDS at 65°C. The membranes were exposed to Kodak X-OMAT XAR-5 autoradiographic film (Sigma, St Louis, MO, USA) at −70°C with intensifying screens for 48–72 h.

*Statistical analysis*

Comparison of the observed genotypic distribution of the *Taq*I hPR alleles between subgroups was carried out, where appropriate, using $\chi^2$ analysis. Calculation of expected genotypic frequencies was performed using the Hardy–Weinberg equation and these expected frequencies were compared by $\chi^2$ analysis, using Yates' correction for expected classes less than 5, with the observed frequencies in each subgroup (Ayala and Kiger, 1980).

### Results

*Characterisation of* Taq*I polymorphism*

Figures 2–4 show representative Southern hybridisation analyses of peripheral leucocyte genomic DNA using *Taq*I as the restriction enzyme and the region-specific cDNA fragments hPR-1, hPR-2 and hPR-3 respectively as the probes (Figure 1b–d). A single, two-allele polymorphism was detected, comprising T1, represented by an estimated 2.7 kb fragment, and T2, represented by an estimated 1.9 kb fragment, and characterised by possession of an additional *Taq*I restriction site with respect to T1. Three genotypes were detected: 2.7 kb/2.7 kb, individuals homozygous for the T1 allele (Figure 2, lanes 1 and 2); 2.7 kb/1.9 kb, individuals heterozygous for the T1 and T2 alleles (Figure 2, lane 3); and 1.9 kb/1.9 kb individuals homozygous for the T2 allele (Figure 3, lanes 1 and 2).

*Localisation of additional* Taq*I restriction site*

hPR cDNA region specific probes were used for a preliminary localisation of the additional *Taq*I site in the hPR gene. Figure 2 shows three *Taq*I-digested genomic DNA samples probed with the hPR-1 cDNA probe (Figure 1b). The restriction fragment hybridisation pattern shows four invariant bands at approximately 5.0, 4.2, 3.5 and 0.6 kb and the two variant fragments at 2.7 kb and 1.9 kb. Figure 3 shows the autoradiographic pattern when the hPR-2 cDNA probe (Figure 1c) was used. As shown, the 1.9 kb fragment

(representing the T2 allele) hybridised with the hPR-2 cDNA probe, indicating the presence of the extra *Taq*I site 3′ to nucleotide position 2593 bp (numbered from ATG1). Figure 4 shows four *Taq*I-digested samples probed with the hPR-3 cDNA probe (Figure 1d). As shown, neither the 2.7 kb nor the 1.9 kb fragment was detectable by this probe: the 0.6 kb fragment was detected, however, indicating that the additional *Taq*I site lies 5′ to 3133 bp (numbered from ATG1). When the hPR-2 cDNA probe (Figure 1c) was used, the 1.9 kb fragment co-segregated with an inconsistently hybridising smaller fragment of 0.8–1.0 kb, suggesting that the latter fragment is composed of predominantly intron G sequence and has limited sequence overlap with the hPR-2 cDNA probe compared with the 1.9 kb fragment. These data are consistent with the location of an additional *Taq*I site in intron G of the T2 hPR allele generating, upon *Taq*I digestion, a 1.9 kb fragment which has extensive sequence overlap with the hPR-2 cDNA probe, and a smaller fragment composed of predominantly intron G sequence.

It is notable that the numbers of restriction fragments hybridising to each of the three cDNA probes hPR-1, hPR-2 and hPR-3 are consistent with the location of the *Taq*I cDNA restriction sites according to Kastner *et al.* (1990) in the human progesterone receptor cDNA (Figure 1).

**Figure 2** Representative Southern analysis of *Taq*I-digested genomic DNA hybridised with the hPR-1 cDNA probe. Lanes 1 and 2 contain samples homozygous for the 2.7 kb T1 allele. Lane 3 contains a sample heterozygous for the T1 and T2 alleles.

**Figure 3** Representative autoradiographic pattern using the hPR-2 cDNA fragment to probe *Taq*I-digested genomic DNA. Three genotypes are evident: 2.7 kb/1.9 kb, individuals heterozygous for the T1 and T2 alleles (lanes 4, 5 and 7); 1.9 kb/1.9 kb, individuals homozygous for T2 (lanes 1 and 2); and 2.7 kb/2.7 kb, individuals homozygous for T1 (lane 6). Lane 3 was empty.

### *Allelic distribution and frequency in ovarian cancer patients and controls*

Significant differences in the distribution of T1 and T2 alleles were observed between the pooled German/Irish ovarian carcinoma cases and in the pooled controls ($P<0.025$) (Table I). No significant difference could be demonstrated in the distribution of the alleles between cases and controls in Ireland ($P=0.18$). The frequency of the T2 allele could not be shown to differ significantly ($P>0.4$) between the case groups of both countries, but was significantly higher in the Irish control group than in the German control group

**Figure 4** Localisation of the additional *Taq*I site in the hPR gene. Lanes 1–4 contain genomic DNA samples heterozygous for the T1 and T2 *Taq*I hPR alleles. The samples were hybridised with the hPR-3 cDNA probe. Differences in band intensity are due to variations in the amount of DNA loaded.

**Table I** Distribution and frequencies of the hPR gene *Taq*I alleles in pooled, Irish and German ovarian cancer case and control groups

| Group | Subgroup | n[a] | n | *T1/T1* Frequency (95% CI)[b] | n | *Genotype T1/T2* Frequency (95% CI) | n | *T2/T2* Frequency (95% CI) | *T2 frequency*[c] (95% CI) |
|---|---|---|---|---|---|---|---|---|---|
| Pooled | Cases[d] | 67 | 43 | 64 (52–75) | 23 | 35 (24–47) | 1 | 1 (0–4) | 0.19 (0.12–0.25) |
| | Controls[d] | 184 | 146 | 79 (73–85) | 33 | 18 (13–23) | 5 | 3 (0–5) | 0.12 (0.09–0.14) |
| Irish | Cases | 41 | 26 | 63.5 (47–78) | 15 | 36.5 (22–53) | 0 | 0 (0–0) | 0.18 (0.10–0.28) |
| | Controls[e] | 83 | 58 | 70 (60–80) | 21 | 25.5 (16–34) | 4 | 5 (1–9) | 0.17 (0.12–0.23) |
| German | Cases | 26 | 17 | 65 (46–85) | 8 | 31 (12–50) | 1 | 4 (0–11) | 0.19 (0.08–0.30) |
| | Controls[e] | 101 | 88 | 88 (80–93) | 12 | 12 (5–18) | 1 | 1 (0–3) | 0.07 (0.03–0.1) |

[a]Number of individuals. [b]Numbers in parentheses are 95% confidence intervals. [c]Allele frequency based on Hardy–Weinberg equation, $p^2+2pq+q^2=1$, where $p$ = frequency of allele T1 and $q$ = frequency of allele T2 in a given subgroup. Hardy–Weinberg distributions were calculated from values of $p$ and $q$ obtained. $\chi^2$ analysis was then used to compare these expected distributions with those observed. All subgroups followed the expected distribution. [d,e]Genotypic distributions of subgroups with common superscripts differed significantly when compared by $\chi^2$ analysis: [d]$P<0.025$, [e]$P<0.02$.

($P<0.02$). The distribution of the two alleles in each group exhibited Mendelian distribution as predicted by the Hardy-Weinberg equation, except in the pooled controls, in which significant deviation from that predicted was approached ($P<0.15$).

### Discussion

Region-specific analysis of the hPR gene demonstrated the presence of an ovarian carcinoma-associated germline RFLP in intron G in the hormone-binding domain-encoding region of the gene. The presence of multiple forms of the progesterone receptor in ovarian carcinoma has been reported (Scharl *et al.*, 1991). Possible explanations include multiple polyadenylation sites, differences in gene regulatory regions or alternative intron splicing. It may be reasonably speculated that the polymorphism we have localised to intron G of the hPR gene has consequences for the integrity of the regulatory functions of the hormone-binding domain, possibly through premature termination of the progesterone receptor mRNA transcript or other faults in the splicing mechanism. Hormone binding and regulated transcriptional activation by the progesterone receptor are dependent upon the presence of a complete, intact hormone-binding domain: deletion of part of this region induces a total loss of hormone-binding and the ability to activate transcription *in vitro* (Dobson *et al.*, 1989).

Although the precise nature of the polymorphism we have identified is yet to be determined, another contingency is that it is a neutral polymorphism co-segregating with other progesterone receptor gene aberrations as yet undetected, as has been suggested in the case of an intron A polymorphism in the oestrogen receptor gene in breast cancer (Yaich *et al.*, 1992).

The demonstration of the tumour-suppressive properties of chromosome 11 in an ovarian cancer cell line (Cao *et al.*, 1993) as well as loss of heterozygosity in ovarian carcinoma (Foulkes *et al.*, 1993) at the hPR gene locus 11q22-q23 (Rousseau-Merck *et al.*, 1987; Mattei *et al.*, 1988) raise the question of a possible tumour-suppressive role for the hPR. The 11q22-q24 locus contains a tumour-suppressor gene relevant to breast cancer (Stickland *et al.*, 1992) and cervical carcinoma (Hampton *et al.*, 1994). Our data give credence to the possibility of a tumour-suppressive role for the hPR gene in ovarian carcinoma.

Considering the hypothesis of Knudson (1971) that one defective copy of a tumour-suppressive gene is inherited and the other subject to a somatic mutation, the over-representation of the T1/T2 heterozygote in ovarian carcinoma patients suggests a possible scenario: that these individuals have inherited a defective copy of the hPR gene and that the other is subject to a somatic mutation. Long-term prospective studies will be required to monitor the incidence of ovarian carcinoma in the control group, especially in those individuals with the T2/T2 genotype.

Whereas the frequency of the T2 allele is similar in the Irish and German case groups, the higher frequency of T2 in the Irish controls led to a smaller difference in T2 frequency between the Irish case and control groups, hence significance could not be established in this population in this study. The fact that the distribution of the *Taq*I RFLP alleles approaches significant deviation from that predicted by the Hardy-Weinberg equation ($P<0.15$) in the pooled controls will require further investigation.

To conclude, the current data show an association between a *Taq*I RFLP and incidence of ovarian carcinoma. This polymorphism is associated with ovarian cancer at a germline level, as opposed to the tumour level. Further work will characterise its role in the development of ovarian carcinoma.

### Acknowledgements
The authors wish to thank Gerry Killeen for reading the manuscript and Don Collins for photography. Financial support for this work was provided by a United Kingdom-Ireland Postgraduate Exchange Scholarship held by NJMcK, an Institutional Grant from the University of Ulm held by DGK, and by American Biogenetic Sciences, USA. Funding was also provided in part by grants from the Health Research Board, Ireland and the Irish Cancer Society to DRH.

### References

AYALA FJ AND KIGER A. (1980). *Modern Genetics*. Benjamin-Cummings: Menlo Park, CA.

CAO Q, CEDRONE E, BARRETT C AND WANG N. (1993). Suppression of *in vitro* growth of ovarian carcinoma cells by microcell-mediated chromosome 11 transfer. *Am. J. Hum. Genet.*, **53**, 1517.

CHADHA S, RAO BR, SLOTMAN BJ, VAN VROONHOVEN CCJ AND VAN DER KWAST TH. (1993). An immunohistochemical evaluation of androgen and progesterone receptors in ovarian tumours. *Hum. Pathol.*, **24**, 90-95.

CLIFFORD-BROUGHAM C. (1989). Human progesterone receptor cDNA; molecular cloning and use in RFLP analysis of human DNA, PhD Thesis. National University of Ireland.

DOBSON ADW, CONNEELY OM, BEATTIE W, MAXWELL BL, MAK P, TSAI M-J, SCHRADER WT AND O'MALLEY BW. (1989). Mutational analysis of the chicken progesterone receptor. *J. Biol. Chem.*, **264**, 4207-4211.

FOULKES WD, CAMPBELL IG, STAMP GW AND TROWSDALE J. (1993). Loss of heterozygosity and amplification on chromosome 11q in human ovarian cancer. *Br. J. Cancer*, **67**, 268-273.

FUQUA SAW, HILL SM, CHAMNESS GC, BENEDIX MG, GREENE GL, O'MALLEY BW AND MCGUIRE WL. (1991). Progesterone receptor gene restriction fragment length polymorphism in human breast tumors. *J. Natl Cancer Inst.*, **83**, 1157-1160.

HAMPTON GM, PENNY LA, BAERGEN RN, LARSON A, BREWER C, LIAO S, BUSBY-EARLE RM, WILLIAMS AWR, STEEL CM, BIRD CC, STANBRIDGE EJ AND EVANS GA. (1994). Loss of heterozygosity in cervical carcinoma: subchromosomal localization of a putative tumour suppressor gene to chromosome 11q22-24. *Proc. Natl Acad. Sci. USA*, **91**, 6953-6957.

HOLLSTEIN M, SIDRANSKY D, VOGELSTEIN B AND HARRIS C. (1991). p53 mutations in human cancers. *Science*, **253**, 49-53.

KASTNER P, KRUST A, TURCOTTE B, STROPP U, TORA L, GRONEMEYER H AND CHAMBON P. (1990). Two distinct estrogen-regulated promoters generate transcripts encoding the two functionally different human progesterone receptor forms A and B. *EMBO J.*, **9**, 1603-1614.

KIEBACK DG, RUNNEBAUM IB, MOEBUS VJ, KREIENBERG R, MCCAMANT SK, EDWARDS CL, JONES LA, TSAI M-J AND O'MALLEY BW. (1993). Chicken ovalbumin upstream promoter transcription factor (COUP-TF): an orphan steroid receptor with a specific pattern of differential expression in human ovarian cancer cell lines. *Gynecol. Oncol.*, **51**, 167-170.

KNUDSON AG. (1971). Mutation and cancer: statistical study of retinoblastoma. *Proc. Natl Acad. Sci. USA*, **68**, 820-823.

MATTEI MG, KRUST A, STROPP U, MATTEI J-F AND CHAMBON P. (1988). Assignment of the human progesterone receptor gene to the q22 band of chromosome 11. *Hum. Genet.*, **78**, 96-97.

MILLER SA, DYKES DD AND POLESKY HF. (1988). A simple salting-out procedure for extracting DNA from human nucleated cells. *Nucleic Acids Res.*, **16**, 315.

MISRAHI M, ATGER M, D'AURIOL L, LOOSFELT H, MERIEL C, FRIDLANSKY F, GUIOCHON-MANTEL A, GALIBERT F AND MILGROM E. (1987). Complete amino acid sequence of the human progesterone receptor cDNA deduced from cloned cDNA. *Biochem. Biophys. Res. Commun.*, **143**, 740-748.

MISRAHI M, VENENCIE P-Y, SAUGIER-VEBER P, SAR S, DESSEN P AND MILGROM E. (1993). Structure of the human progesterone receptor gene. *Biochim. Biophys. Acta*, **1216**, 289-292.

NOGUCHI T, KITAWAKI J, TAMURA T, KIM T, KANNO H, YAMAMOTO T AND OKADA H. (1993). Relationship between aromatase activity and steroid receptor levels in ovarian tumours from post-menopausal women. *J. Steroid Biochem. Mol. Biol.*, **44**, 657-660.

ROUSSEAU-MERCK MF, MISRAHI M, LOOSFELT H, MILGROM E AND BERGER R. (1987). Localization of the human progesterone receptor gene to chromosome 11q22-q23. *Hum. Genet.*, **77**, 280-282.

SANDY MS, CHIOCCA SM AND CERUTTI PA. (1992). Genotypic analysis of mutations in *Taq*I restriction recognition sites by restriction fragment length polymorphism/polymerase chain reaction. *Proc. Natl Acad. Sci. USA*, **89**, 890-894.

SCHARL A, LORINCZ MA, GREENE GL AND HOLT JA. (1991). Ovarian carcinomas express several forms of the progesterone receptor. *Arch. Gynecol. Obstet.*, **250,** 181-182.

SCULLY SR. (1987). Pathology of ovarian carcinoma. In *Ovarian Malignancies*, Piver SM (ed.) pp. 72-95. Churchill Livingstone: Edinburgh.

SLOTMAN BJ, NAUTA JJ AND RAMANATH-RAO BR. (1990). Survival of patients with ovarian cancer: apart from stage and grade, tumour progesterone receptor content is a prognostic indicator. *Cancer*, **66,** 740-744.

STICKLAND JE, TOMLINSON IP, LEE AS, EVANS MF AND McGEE JO. (1992). Allelic loss on chromosome 11q is a frequent event in breast cancer (abstract). *Br. J. Cancer*, **66** (Suppl. XVII), 3.

WANLESS C, BARKER S, PUDDEFOOT JR, PANAHY C, GOODE AW, VINSON GP AND PHILLIPS IR. (1991). Somatic change in the estrogen receptor gene associated with altered expression of the progesterone receptor. *Anticancer Res.*, **11,** 139-142.

YAICH L, DUPONT WD, CAVENER DR AND PARL F. (1992). Analysis of the *Pvu*II restriction fragment length polymorphism and exon structure of the estrogen receptor gene in breast cancer and peripheral blood. *Cancer Res.*, **52,** 77-83.


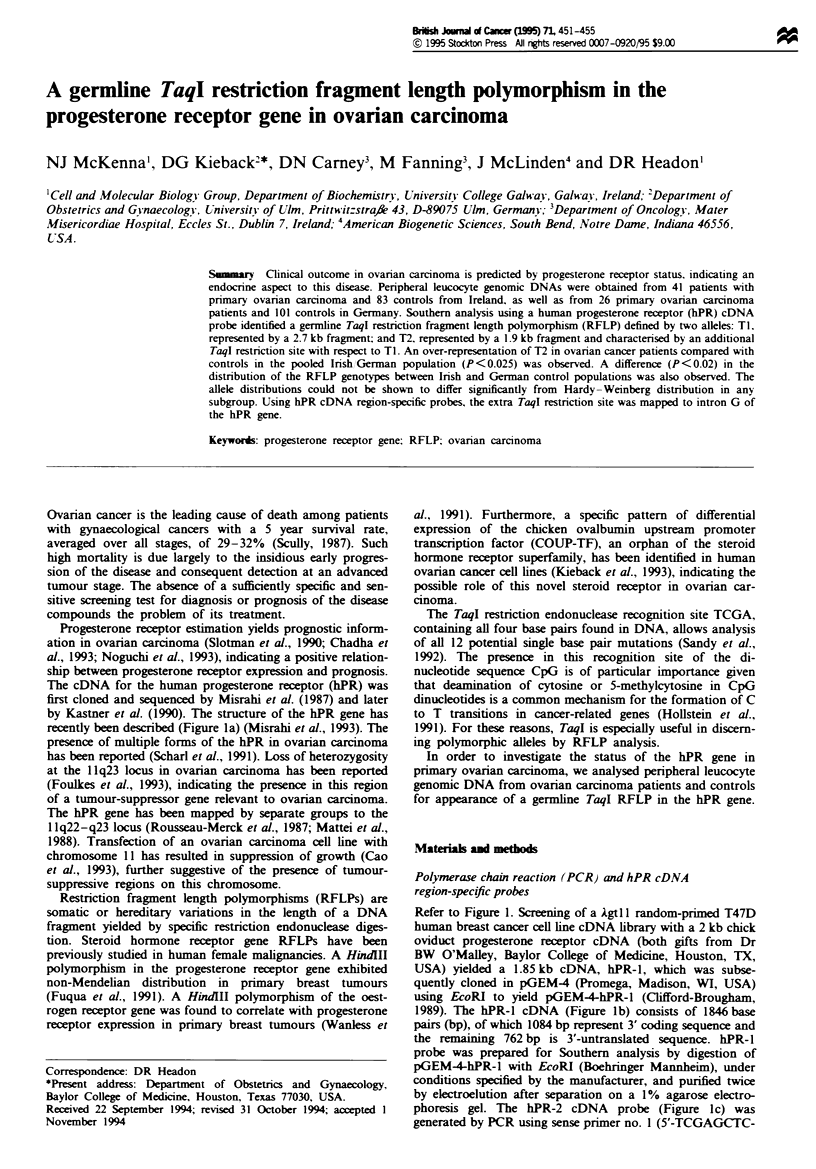

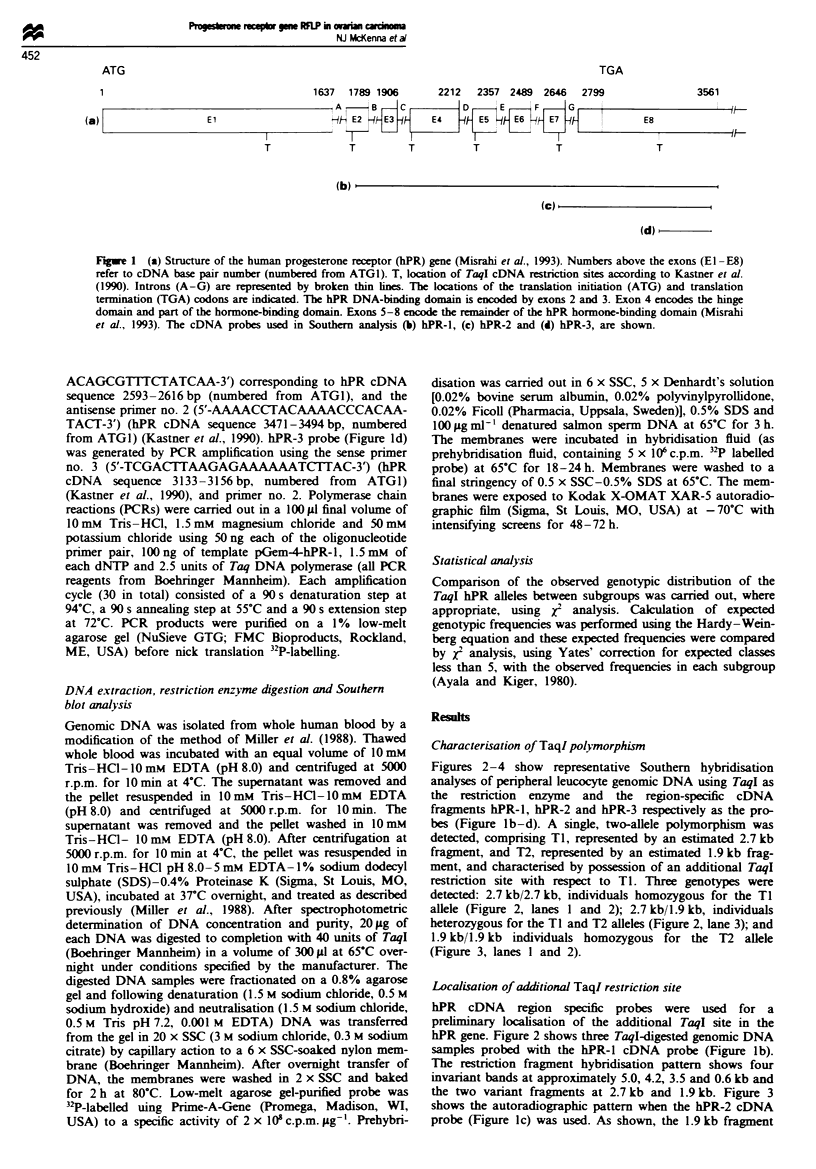

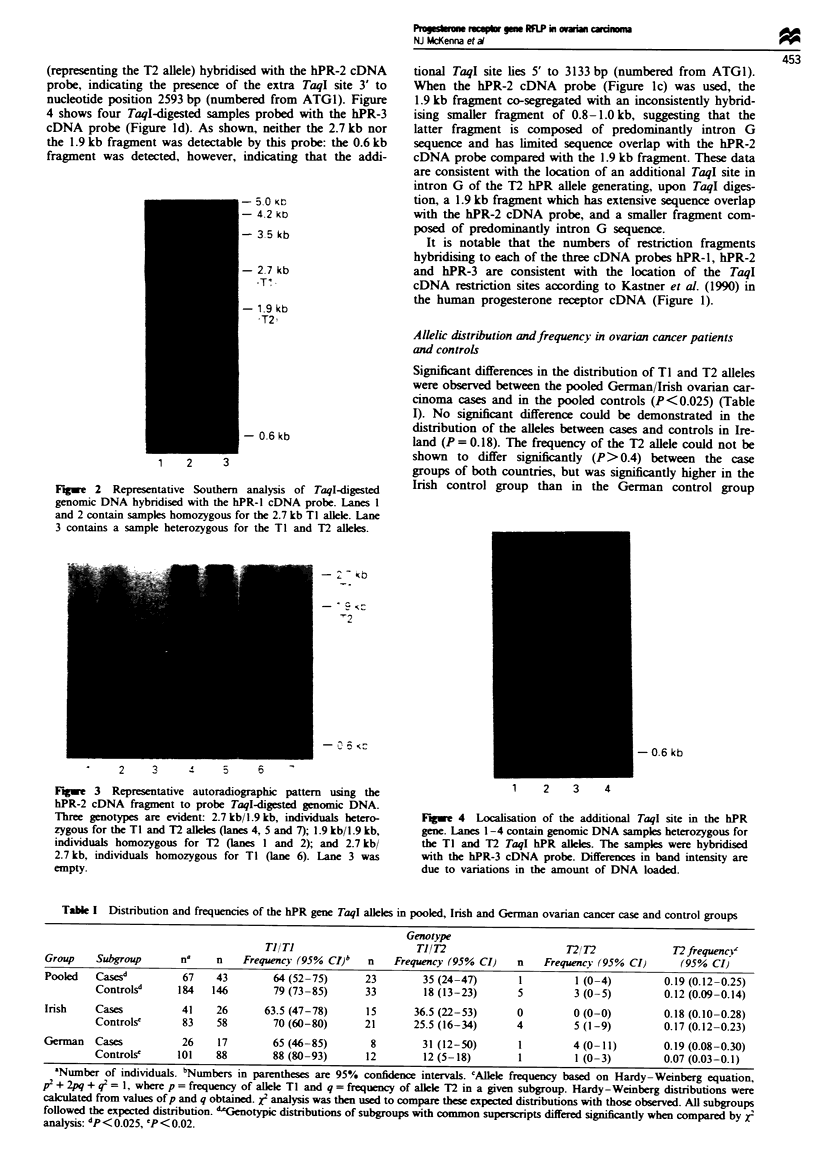

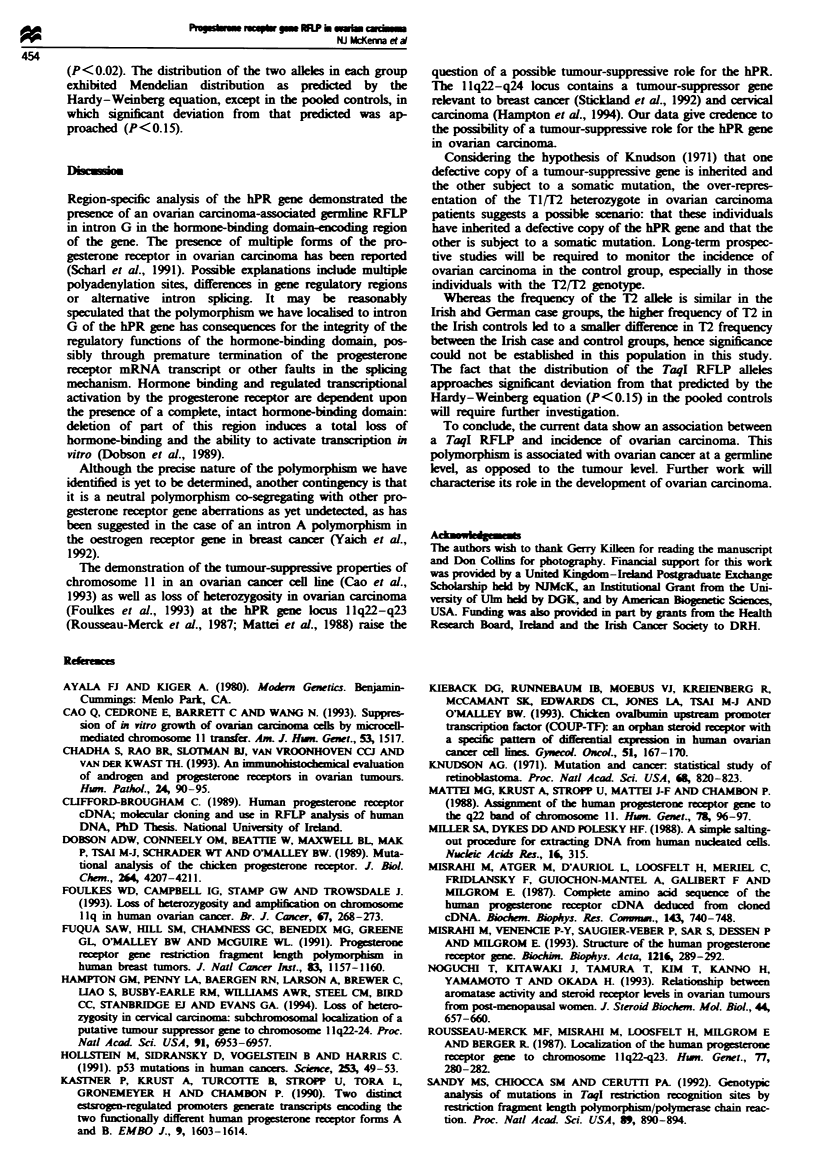

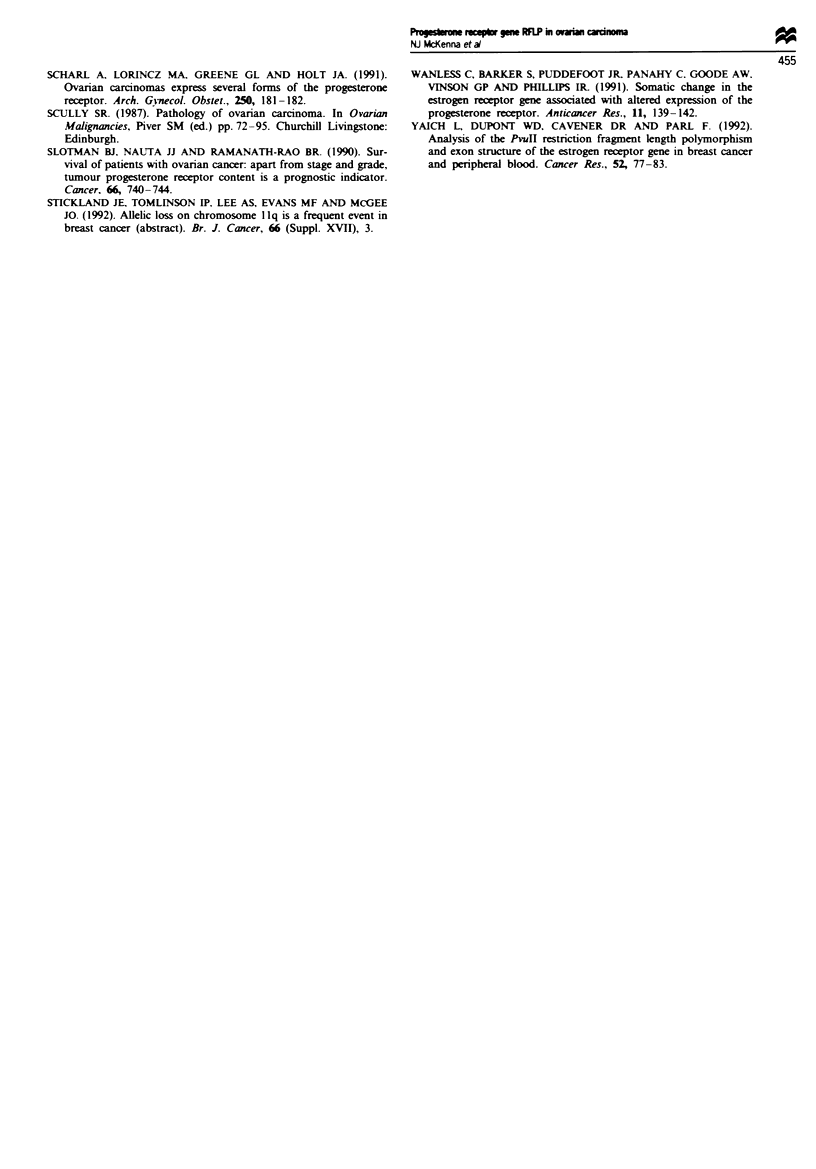


## References

[OCR_00504] Chadha S., Rao B. R., Slotman B. J., van Vroonhoven C. C., van der Kwast T. H. (1993). An immunohistochemical evaluation of androgen and progesterone receptors in ovarian tumors.. Hum Pathol.

[OCR_00514] Dobson A. D., Conneely O. M., Beattie W., Maxwell B. L., Mak P., Tsai M. J., Schrader W. T., O'Malley B. W. (1989). Mutational analysis of the chicken progesterone receptor.. J Biol Chem.

[OCR_00520] Foulkes W. D., Campbell I. G., Stamp G. W., Trowsdale J. (1993). Loss of heterozygosity and amplification on chromosome 11q in human ovarian cancer.. Br J Cancer.

[OCR_00524] Fuqua S. A., Hill S. M., Chamness G. C., Benedix M. G., Greene G. L., O'Malley B. W., McGuire W. L. (1991). Progesterone receptor gene restriction fragment length polymorphisms in human breast tumors.. J Natl Cancer Inst.

[OCR_00530] Hampton G. M., Penny L. A., Baergen R. N., Larson A., Brewer C., Liao S., Busby-Earle R. M., Williams A. W., Steel C. M., Bird C. C. (1994). Loss of heterozygosity in cervical carcinoma: subchromosomal localization of a putative tumor-suppressor gene to chromosome 11q22-q24.. Proc Natl Acad Sci U S A.

[OCR_00540] Hollstein M., Sidransky D., Vogelstein B., Harris C. C. (1991). p53 mutations in human cancers.. Science.

[OCR_00546] Kastner P., Krust A., Turcotte B., Stropp U., Tora L., Gronemeyer H., Chambon P. (1990). Two distinct estrogen-regulated promoters generate transcripts encoding the two functionally different human progesterone receptor forms A and B.. EMBO J.

[OCR_00552] Kieback D. G., Runnebaum I. B., Moebus V. J., Kreienberg R., McCamant S. K., Edwards C. L., Jones L. A., Tsai M. J., O'Malley B. W. (1993). Chicken ovalbumin upstream promoter transcription factor (COUP-TF): an orphan steroid receptor with a specific pattern of differential expression in human ovarian cancer cell lines.. Gynecol Oncol.

[OCR_00559] Knudson A. G. (1971). Mutation and cancer: statistical study of retinoblastoma.. Proc Natl Acad Sci U S A.

[OCR_00565] Mattei M. G., Krust A., Stropp U., Mattei J. F., Chambon P. (1988). Assignment of the human progesterone receptor to the q22 band of chromosome 11.. Hum Genet.

[OCR_00576] Misrahi M., Atger M., d'Auriol L., Loosfelt H., Meriel C., Fridlansky F., Guiochon-Mantel A., Galibert F., Milgrom E. (1987). Complete amino acid sequence of the human progesterone receptor deduced from cloned cDNA.. Biochem Biophys Res Commun.

[OCR_00582] Misrahi M., Venencie P. Y., Saugier-Veber P., Sar S., Dessen P., Milgrom E. (1993). Structure of the human progesterone receptor gene.. Biochim Biophys Acta.

[OCR_00588] Noguchi T., Kitawaki J., Tamura T., Kim T., Kanno H., Yamamoto T., Okada H. (1993). Relationship between aromatase activity and steroid receptor levels in ovarian tumors from postmenopausal women.. J Steroid Biochem Mol Biol.

[OCR_00595] Rousseau-Merck M. F., Misrahi M., Loosfelt H., Milgrom E., Berger R. (1987). Localization of the human progesterone receptor gene to chromosome 11q22-q23.. Hum Genet.

[OCR_00598] Sandy M. S., Chiocca S. M., Cerutti P. A. (1992). Genotypic analysis of mutations in Taq I restriction recognition sites by restriction fragment length polymorphism/polymerase chain reaction.. Proc Natl Acad Sci U S A.

[OCR_00621] Slotman B. J., Nauta J. J., Rao B. R. (1990). Survival of patients with ovarian cancer. Apart from stage and grade, tumor progesterone receptor content is a prognostic indicator.. Cancer.

[OCR_00630] Wanless C., Barker S., Puddefoot J. R., Panahy C., Goode A. W., Vinson G. P., Phillips I. R. (1991). Somatic change in the estrogen receptor gene associated with altered expression of the progesterone receptor.. Anticancer Res.

[OCR_00636] Yaich L., Dupont W. D., Cavener D. R., Parl F. F. (1992). Analysis of the PvuII restriction fragment-length polymorphism and exon structure of the estrogen receptor gene in breast cancer and peripheral blood.. Cancer Res.

